# New York College Veterinary Surgeons

**Published:** 1893-12

**Authors:** 


### VETERINARY GRADUATES, 1893.

### KANSAS CITY VETERINARY COLLEGE.

*March 17th.—Degree.*

Onesimus G. Atherton

William G. Hawkey

Joseph Pott

Charles Saunders

### NEW YORK COLLEGE VETERINARY SURGEONS.

*Degree V. S. (Veterinary Surgeon.)*

| | |
|---|---|
| Henry Amling, Jr. | N. Y. City |
| Ira K. Atherton | Arrowsmith, Illinois |
| William Cook | New York City, N. Y. |
| Charles Doerrie | Salisbury, Mo. |
| William J. Finn | Brooklyn, N. Y. |
| Herbert B. Hamilton | Boston, Mass. |
| Robert C. Helmer | Scranton, Pa. |
| Alexander Johnston | New York |
| Robert E. Jones | New York City, N. Y. |
| James H. Kelley | New Haven, Ct. |
| Harry W. Kornobis | Brooklyn, N. Y. |
| Dennis McAuliffe | New York City, N. Y. |
| Albert G. Mehrhoff | Little Ferry, N. J. |
| Irwin C. Newhard | Allentown, Pa. |
| Thomas F O'Dea | Ghent, N. Y. |
| Edward A. Paul | Brooklyn, N. Y. |
| W. L. Sturges | North Norwich, N. Y. |
| Isaac White | New York City, N. Y. |
| Fraley E. Winslow | Whitestone, N. Y. |
| Louis J. Wolfort | St. Louis, Mo. |
| Addison R. Wiley | Windsor, N. J. |

